# Arsenic Trioxide Impacts Viral Latency and Delays Viral Rebound after Termination of ART in Chronically SIV‐Infected Macaques

**DOI:** 10.1002/advs.201900319

**Published:** 2019-05-07

**Authors:** Qing Yang, Fengling Feng, Pingchao Li, Enxiang Pan, Chunxiu Wu, Yizi He, Fan Zhang, Jin Zhao, Ruiting Li, Liqiang Feng, Fengyu Hu, Linghua Li, Huachun Zou, Weiping Cai, Thomas Lehner, Caijun Sun, Ling Chen

**Affiliations:** ^1^ State Key Laboratory of Respiratory Disease Guangzhou Institutes of Biomedicine and Health (GIBH) Chinese Academy of Sciences Guangzhou 510530 China; ^2^ School of Public Health (Shenzhen) Sun Yat‐sen University Guangdong 518107 China; ^3^ Guangzhou Eighth People's Hospital Guangzhou Medical University Guangzhou 510182 China; ^4^ Mucosal Immunology Unit King's College London at Guy's Hospital London WC2R 2LS UK

**Keywords:** arsenic trioxide, antiretroviral therapy (ART), functional cures, human immunodeficiency virus‐1 (HIV‐1), latency, Simian immunodeficiency virus (SIV)

## Abstract

The latent viral reservoir is the source of viral rebound after interruption of antiretroviral therapy (ART) and is the major obstacle in eradicating the latent human immunodeficiency virus‐1 (HIV‐1). In this study, arsenic class of mineral, arsenic trioxide, clinically approved for treating acute promyelocytic leukemia, is demonstrated to reactivate latent provirus in CD4+ T cells from HIV‐1 patients and Simian immunodeficiency virus (SIV)‐infected macaques, without significant systemic T cell activation and inflammatory responses. In a proof‐of‐concept study using chronically SIVmac239‐infected macaques, arsenic trioxide combined with ART delays viral rebound after ART termination, reduces the integrated SIV DNA copies in CD4+ T cells, and restores CD4+ T cells counts in vivo. Most importantly, half of arsenic trioxide‐treated macaques show no detectable viral rebound in the plasma for at least 80 days after ART discontinuation. Mechanistically, the study reveals that CD4 receptors and CCR5 co‐receptors of CD4+ T cells are significantly downregulated by arsenic trioxide treatment, which reduces susceptibility to infection after provirus reactivation. Furthermore, an increase in SIV‐specific immune responses after arsenic trioxide treatment may contribute to suppression of viral rebound. This work suggests that arsenic trioxide in combination with ART is a novel regimen in down‐sizing or even eradicating latent HIV‐1 reservoir.

Antiretroviral therapy (ART) results in effective control of human immunodeficiency virus‐1 (HIV‐1) replication in plasma to undetectable levels, but fails to eradicate latent viral reservoir, which has been recognized as the major source of viral rebound after ART discontinuing.^1^, ^2^, ^3^ Recent studies demonstrated that viral reservoirs were seeded within 1–3 days of infection in the simian immunodeficiency virus (SIV) model of acquired immune deficiency syndrome (AIDS), and therefore even early ART treatment after 3 days of infection cannot block the establishment of persistent infection.^4^, ^5^, ^6^ Alternatively, several strategies including shock‐and‐kill, chimeric antigen receptor T‐cell therapy, therapeutic vaccination, and gene editing^7^, ^8^, ^9^, ^10^, ^11^, ^12^ are extensively explored to target the latent reservoir.

It is therefore of great interest to develop novel strategies to render latent proviruses susceptible to eradication. Among them, reactivation of latent proviruses with latency‐reversing agents (LRA) is critical for the efficacy of shock‐and‐kill strategy. An ideal LRA should activate the latent proviruses without extensive T cell activation and inflammatory responses. Various compounds, including chromatin remodeling agents such as histone deacetylase acetylation inhibitors (suberoylanilide hydroxamic acid (SAHA) and valproic acid (VPA)) or T cell activators (bryostatin‐1), have been tested as potential LRA to “shock out” the latent proviruses in preclinical and clinical studies.^13^, ^14^, ^15^, ^16^ However, a safe and effective LRA has so far not been identified for clinical use.

Arsenic class of mineral has long history of being used in medical practice. In traditional Chinese medicine, it is well known as “Pi‐Shuang” in the treatment of tuberculosis and parasitic infections in ancient China, which was documented in Shennong Bencao Jing (Shennong Emperor's Classic of Materia Medica) in the 1st century AD. In modern medicine, an arsenical drug, arsenic trioxide (As_2_O_3_), has been approved for the treatment of acute promyelocytic leukemia (APL) and hepatocellular carcinoma (HCC) in clinic.^17^, ^18^, ^19^ Several studies demonstrated that arsenic trioxide can react with cysteine residues and elicit critical transcription signal factors, including protein kinase C, NF‐kB, promyelocytic leukemia nuclear body (PML‐NB), mitogen‐activated protein kinases, and retinoic acid receptor alpha.^20^, ^21^ Arsenic can also irreversibly inhibit mammalian thioredoxin reductase which is one of the electron donor systems that control cellular proliferation, viability, and apoptosis through elevated oxidative stress.^22^ Since these transcription factors and signal pathways were reported to affect HIV‐1 latency, arsenic trioxide might also modulate HIV‐1 reservoir. For example, arsenic trioxide was reported to degrade PML‐NB suppressor by sumoylation and thereby reverse the inhibition of transcription, and the silenced but transcriptionally competent HIV‐1 proviruses usually reside in close proximity to PML‐NB in CD4+ T cells.^23^ Indeed, previous studies have shown that arsenic trioxide enhanced retroviral reverse transcription of HIV‐1^24^ and induced activation of latent HIV‐1 in Jurkat T cell line.^23^, ^25^ Furthermore, sodium arsenite reactivated gene expression and virus replication from the latency genome of herpes simplex virus type 1 in vitro.^26^ Nevertheless, there is no evidence whether the latent HIV/SIV reservoir can be targeted following arsenic trioxide administration in vivo.

Collectively, these studies inspired us to explore arsenic trioxide in combination with ART as a novel regimen to target the viral reservoir for HIV‐1 functional cure. In this study, we therefore investigated how viral reservoir could be regulated by arsenic trioxide in primary CD4+ T lymphocytes and in SIV‐infected, ART‐treated Chinese rhesus macaques.

The first objective of this study was to find out whether arsenic trioxide has any effects on the latent provirus, and we found that arsenic trioxide induced HIV‐1 reactivation in a dose‐dependent manner in Jurkat‐Lat HIV‐1 full length clone A10.6 cells (**Figure** **1**A,B). After 24 h treatment with different concentrations of arsenic trioxide, the percentage of green fluorescent protein (GFP)‐expressing cells, which represents the transcriptional activated HIV‐1, increased from 2.8% to 13.2%, when the concentration of arsenic trioxide increased from 125 × 10^−9^ to 10 × 10^−6^ m (Figure 1B). A histone deacetylase inhibitor VPA (5 × 10^−3^ m) upregulated GFP‐expressing cells to 26.7%, and was enhanced to 56% with added 1.25 × 10^−6^ m arsenic trioxide (Figure 1C). This demonstrated that arsenic trioxide synergistically reactivated latent HIV‐1 when combined with VPA (Figure 1A–C).

**Figure 1:**
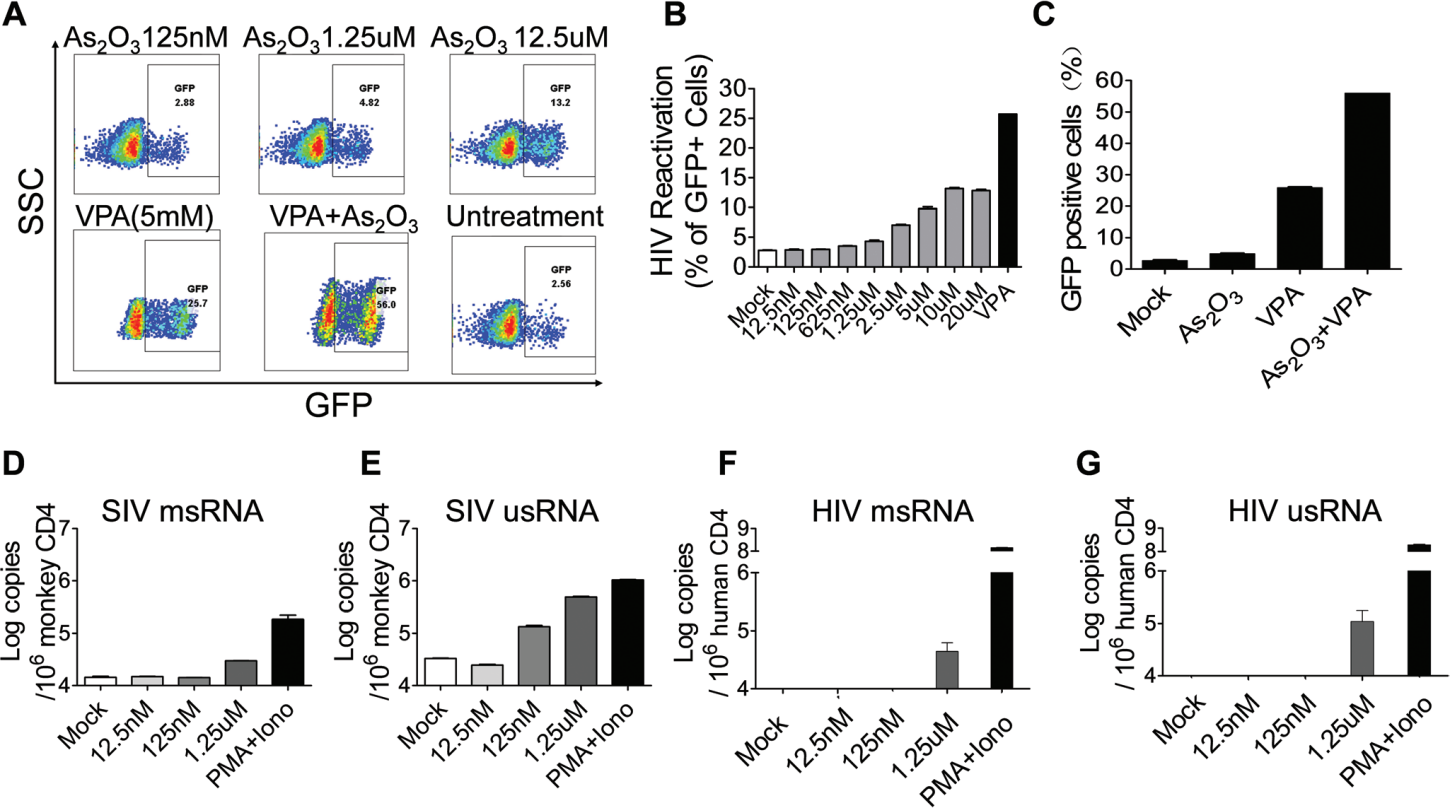
Arsenic trioxide reactivated latent provirus in J‐Lat HIV latency cell line, primary CD4+ T cells from HIV‐1 patient, and SIVmac239‐infected macaques. A) J‐Lat A10.6 cells were treated with arsenic trioxide or VPA. At 24 h post‐treatment, the percentage of GFP‐positive cells was analyzed by flow cytometry. GFP expression represents the transcriptional activity of HIV‐1 promoter. VPA was used as a positive control for provirus reactivation. B) Dose‐dependent reactivation of HIV‐1 latency by arsenic trioxide in J‐Lat A10.6 cells. C) Synergistic activation of HIV‐1 latency by arsenic trioxide with VPA in J‐Lat A10.6 cells. The cell‐associated RNA (CA‐ RNA) expression in primary CD4+ T cells from SIV‐infected rhesus macaques and HIV‐1‐infected patients were measured as our previously reported.^29^ The numbers of D) SIV msRNA, E) SIV usRNA, and F) HIV‐1 msRNA, G) HIV‐1 usRNA per million of CD4+ T cells were determined by nested PCR. Data were presented as the mean ± standard deviation of triplicate experiments. SSC: Side Scatter; GFP: green fluorescent protein; VPA: valproic acid; RM: Rhesus Macaques; msRNA: multiply spliced RNA; usRNA: unspliced RNA.

Although Jurkat‐Lat T cell line is a commonly used model to mimic HIV‐1 latency, it may not reflect the genetic diversity of viral reservoir pool of primary cells, in which HIV proviruses are integrated into different sites in the host genome. Hence, we used primary CD4+ T cells from SIV‐infected rhesus macaques and HIV‐1‐infected patients, to further evaluate reactivation efficacy of arsenic trioxide.

SIV‐infected macaques used in this study had a history of SIVmac239 infection for more than 5 years as previously reported,^27^, ^28^ and had several courses of ART therapy to control viral load in plasma to undetectable levels (Table S1, Supporting Information). In addition, CD4+ T cells were obtained from four HIV‐1 patients who received prolonged ART therapy and achieved an effective control of viral replication in plasma (Table S2, Supporting Information). Purified CD4+ T cells were incubated with arsenic trioxide. Cell‐associated SIV or HIV‐1 RNA in arsenic trioxide‐treated CD4+ T lymphocytes isolated from SIV‐infected macaques or HIV‐1 patients was detected as previously reported.^29^ Cell‐associated RNA in peripheral blood mononuclear cells (PBMC), such as multiply spliced RNA (msRNA) and unspliced RNA (usRNA), was associated with HIV replication and disease progression, and was thought to be a sensitive parameter to represent the status of viral transcription and reactivation. Notably, the level of msRNA and usRNA copies in resting CD4+ T cells from both SIV‐infected macaques (Figure 1D,E) and HIV‐1 patients (Figure 1F,G) were significantly induced with arsenic trioxide in a dose‐dependent manner. These data demonstrated that arsenic trioxide can activate HIV‐1 or SIV transcription in primary CD4+ T cells. To evaluate if arsenic trioxide exerts cell cytotoxicity, viability was assessed in primary PBMCs, treated with increasing concentrations of arsenic trioxide. There were no significant effects on cell viability at concentration as high as 10 × 10^−6^ m, but the toxicity became obvious at 20 × 10^−6^ m and higher concentrations (Figure S1, Supporting Information).

We next investigated the effect of arsenic trioxide on T cell activation, proliferation and inflammation. PBMCs from SIV‐infected macaques were treated with arsenic trioxide (1.25 × 10^−6^–12.5 × 10^−6^ m) for 24 h. The expression of CD25, CD38, CD69, and HLA‐DR on PBMCs was determined by flow cytometry. A cocktail of phorbol 12‐myristate 13‐acetate (PMA) and ionomycin was used as a positive control for T cell activation and proliferation. There was no significant increase in CD25, CD69, or HLA‐DR and CD38 expression in CD4+ T and CD8+ T cells treated with arsenic trioxide for 24 h at concentrations up to 12.5 × 10^−6^ m (**Figure** **2**A–F), unlike those with the PMA stimulation. In addition, there was no significant increase in the proliferative responses of CD4+ T and CD8+ T cells with arsenic trioxide treatment, tested by staining with carboxyfluorescein succinimidyl ester (CFSE) (Figure 2G,H). Similar results were also found when CD4+ T and CD8+ T cells were incubated with arsenic trioxide for 5 days at 12.5 × 10^−6^ m (Figure S2, Supporting Information).

**Figure 2:**
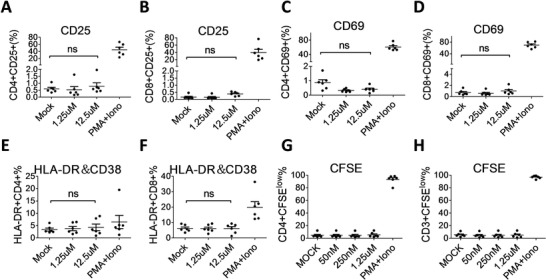
Effects of arsenic trioxide on T cell activation and proliferation. PBMCs from Chinese rhesus macaques were treated with arsenic trioxide for 24 h. A–F) The expressions of T cell activation markers were detected by flow cytometry, including CD25+, CD69+, and CD38+/HLA‐DR+ on the surface of CD8+ T and CD4+ T cells from SIV‐infected macaques. The proliferative responses of G) CD4+ T and H) CD8+ T cells after arsenic trioxide treatment were determined by carboxyfluorescein succinimidyl ester (CFSE) staining. PMA and ionomycin were used as positive controls for T cell activation and proliferation.

Proinflammatory responses, characterized by the production of inflammatory cytokines, such as interleukin (IL)‐1β, IL‐6, IL‐8, tumor necrosis factor alpha (TNF‐α), and interferon (IFN)‐γ, are often increased by traditional LRA treatment. We therefore investigated the effects of arsenic trioxide on the inflammatory responses. Although IL‐1β and IL‐6 were induced, IL‐8 and IFN‐γ were not elicited when PBMCs were treated with arsenic trioxide up to 12.5 × 10^−6^ m (**Figure** **3**A). TNF‐α was increased only at the higher concentration of arsenic trioxide of 12.5 × 10^−6^ m, which might be related to the arsenic trioxide‐induced cell apoptosis. Next, we used lipopolysaccharide (LPS) to stimulate freshly isolated PBMC cells to further investigate the effects of arsenic trioxide on the inflammatory responses. Interestingly, arsenic trioxide appeared to suppress the LPS‐induced expression of IL‐1β, IL‐6, IL‐8, and IFN‐γ (Figure 3B). Taken together, arsenic trioxide can reactivate latent proviruses without significantly devastating T cell activation and proinflammatory responses.

**Figure 3:**
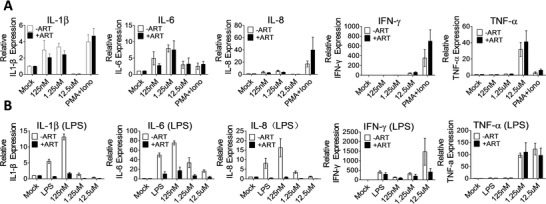
Inflammatory responses after arsenic trioxide treatment. A) PBMCs were treated with arsenic trioxide at the given concentrations for 24 h with or without ART drugs. The expression of the inflammatory cytokines was measured by quantitative RT‐PCR analysis. B) PBMCs in the presence of LPS (5 µg L^−1^) were treated with arsenic trioxide at the given concentrations for 24 h with or without ART drugs; ART containing FTC(3 mg L^−1^) and PMPA(2 mg L^−1^); β‐macroglobulin was used as the internal control. Data are the means ± standard deviations from three independent experiments.

We then assessed the potential therapeutic effects of administering arsenic trioxide in chronically SIVmac239‐infected, ART‐treated rhesus macaques (**Figure** **4**A). The arsenic trioxide formulation used in this study was a clinically available drug NAWEIYA, which has been approved for the treatment of acute promyelocytic leukemia and hepatocellular carcinoma in China. A cohort of chronically SIVmac239‐infected rhesus macaques was divided into two comparable groups according to viral load, weight, and age (Table S1, Supporting Information). One group of macaques received ART alone and another group received in addition intravenous injections of 0.16 mg kg^−1^ arsenic trioxide, which is the same dosage as that clinically used to treat APL patients. All macaques have been infected with SIVmac239 for more than 5 years and had persistent viral infection.^28^, ^29^ They have previously received several courses of ART (30 mg kg^−1^ (*R*)‐9‐(2‐phosphonylmethoxypropyl) adenine (PMPA) and 20 mg kg^−1^ beta‐2,3‐dideoxy‐3‐thia‐5‐fluorocytidine (FTC)). Plasma viral loads in these macaques dropped below the detection limit during ART administration, but rebound quickly after stopping ART (Table S1, Supporting Information). Consistent with our previous observation, the SIV RNA copies rebounded in all of ART alone–treated macaques in an average interval of 22 days following discontinuation of ART (Figure 4C). Importantly, arsenic trioxide in combination with ART therapy delayed virus rebound after ART discontinuation.

**Figure 4:**
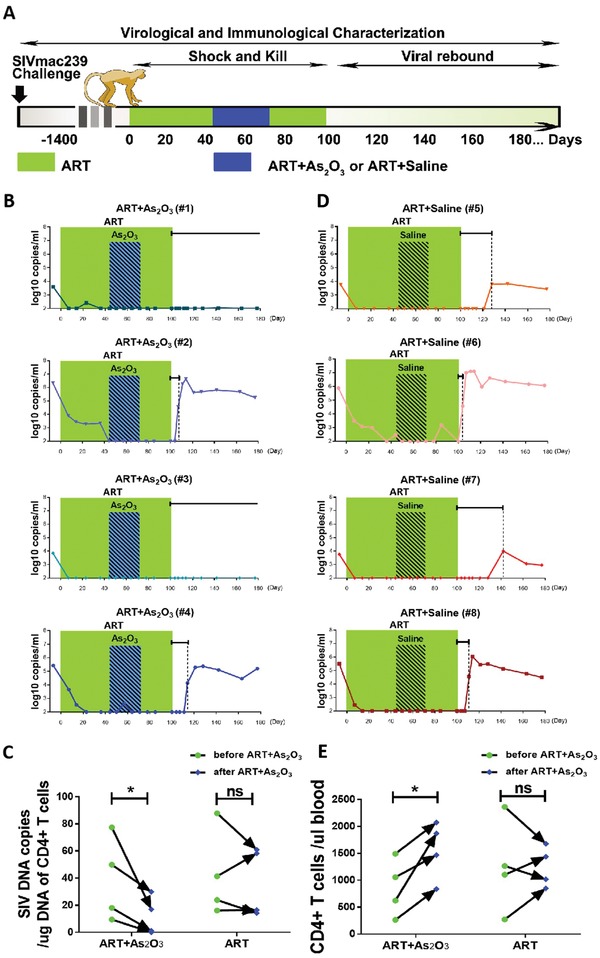
Arsenic trioxide in combination with ART delayed viral rebound in SIV‐infected rhesus macaques. A) Experimental schedule of proof‐of‐concept for functional cure by administration of arsenic trioxide combined with ART in chronic SIV‐infected macaques. Eight Chinese rhesus macaques were divided into two groups according to viral load, weight, and age. One group received ART therapy alone (*n* = 4), and another group received intravenous injections of arsenic trioxide during ART therapy (*n* = 4). B) The viral loads for each experimental macaque in ART+arsenic trioxide groups were monitored overtime by real‐time PCR; the sensitivity of this assay was 100 copies mL^−1^ plasma. C) The viral load for each experimental macaque in ART‐only groups was monitored as above. The bold horizontal line in (B) and (C) represents the time of viral rebound after ART was discontinued. D) Alu‐PCR analysis of the integrated SIV provirus copies in CD4+ T cells before and after ART with or without combination of arsenic trioxide. E) Numbers of circulating CD4+ T lymphocytes before and after ART with or without combination of arsenic trioxide. Cells were determined using BD TruCount tubes. * *p* < 0.05.

Especially, two macaques showed no detectable virus in the plasma for at least 80 days after ART discontinuation (Figure 4B). Although no significant viral blips were observed in those monkeys, a significant decrease of integrated SIV provirus was found in CD4+ T lymphocytes in group of arsenic trioxide in combination with ART therapy, when compared with ART alone (Figure 4D, *p* < 0.05). In addition, the CD4+ T lymphocyte count was significantly restored in macaques with arsenic trioxide administration, but not in the ART alone group (Figure 4E, *p* < 0.05). These findings suggest that arsenic trioxide in combination with ART therapy resulted in reducing the SIV reservoirs, restoring the immune reconstitution in chronically SIV‐infected macaques, and delaying viral rebound after ART discontinuation.

We further explored the mechanism of arsenic trioxide in delaying viral rebound after ART discontinuation. Primary T cells isolated from SIV‐infected or SIV‐uninfected macaques were treated with arsenic trioxide, and results showed that although there was no significant change of the proportion of CD4+ T cells (**Figure** **5**A), the expression level of CD4 and CCR5 on arsenic trioxide‐treated CD4+ T cells was significantly downregulated in a dose‐dependent manner with arsenic trioxide treatment (*p* < 0.001, Figure 5B,C). In contrast, the proportion and expression level of CD8 and CCR5 on CD8+ T cells showed no significant change when treated with arsenic trioxide (Figure S3, Supporting Information).

**Figure 5:**
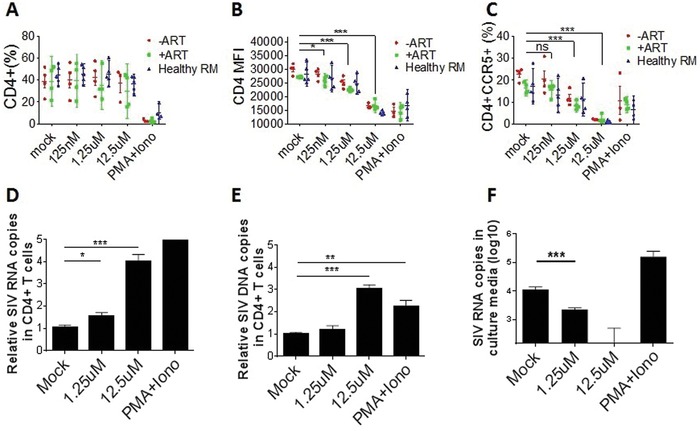
Arsenic trioxide downregulated the expression of CD4 and CCR5 on CD4+ T cells and reduced susceptibility to spread SIV infection. Primary CD4+ T cells isolated from SIV‐infected or SIV‐negative healthy macaques were treated with arsenic trioxide for 5 days and then assessed using flow cytometry. A) the proportion of CD4+ T cells; B) the median fluorescent intensity (MFI) of CD4 expression; C) expression level of CCR5 on the surface of CD4+ T cells. Sorted CD4+ T cells from SIV‐infected macaques were incubated with arsenic trioxide for 72 h, and the D) intracellular viral RNA copies, E) intracellular viral DNA copies, and F) viral RNA copies in the culture media of primary CD4+ T cells were determined by RT‐qPCR. The final data were represented as the means ± standard deviations of triplicate experiments. *p < 0.05, **p < 0.01, ***p < 0.001.

Since CD4 receptors and CCR5 coreceptors are critical for HIV/SIV attachment and entry into target cells, these results suggest that arsenic trioxide might reduce the susceptibility of CD4+ T cells to viral infection during reactivation of latency provirus. To this end, primary CD4+ T cells with productive virions from SIV‐infected rhesus macaques were incubated with different concentrations of arsenic trioxide for 62 h. The level of SIV viral copies in the intracellular extracts and in the culture media of primary CD4+ T cells were then measured. Compared with the mock‐treated samples, a dose‐dependent increase in SIV RNA copies (Figure 5D) and SIV DNA copies (Figure 5E) in CD4+ T cells was found after arsenic trioxide treatment. This was consistent with previous data (Figure 1D,E). However, a dose‐dependent decrease in viral copies in the culture media of primary CD4+ T cells was found with the arsenic trioxide‐treated samples, and undetectable viral copies with 12.5 × 10^−6^ m of arsenic trioxide ((Figure 5F, *p* < 0.001). These findings suggest that arsenic trioxide suppressed SIV spread during reactivation of latent SIV provirus by downregulating the expression of viral CD4 receptors and CCR5 coreceptors.

Finally, we examined the effect of arsenic trioxide on T cell responses in chronically SIV‐infected Chinese rhesus macaques. Specific T cell responses against SIV Gag, Pol, and Env antigens, expressed as IFN‐γ‐mediated enzyme‐linked immunosorbent spot (ELISPOT) responses, were found in all chronic SIV‐infected Chinese rhesus macaques (**Figure** **6**A,B). SIV‐specific T cell responses were significantly increased after treatment with arsenic trioxide in combination with ART (Figure 6C). In comparison, SIV antigen‐specific T cell responses were relatively unchanged in ART‐only treated macaques (Figure 6C). Importantly, in vivo administration of arsenic trioxide in chronic SIV‐infected macaques significantly downregulated the expression of CCR5 coreceptors, with no significant change of expression in CD95 and CD69 T cell activation markers in CD4+ T cells (Figure 6D), which was consistent with the in vitro data (Figure 5). Taken together, these results suggest that arsenic trioxide in combination with ART may regulate antiviral T cell immune responses without devastating systemic T cell activation. The increased SIV‐specific immune responses can eliminate the infected cells harboring latent SIV during the viral reactivation, and thus contribute to suppression of viral rebound in chronic SIV‐infected rhesus macaques.

**Figure 6:**
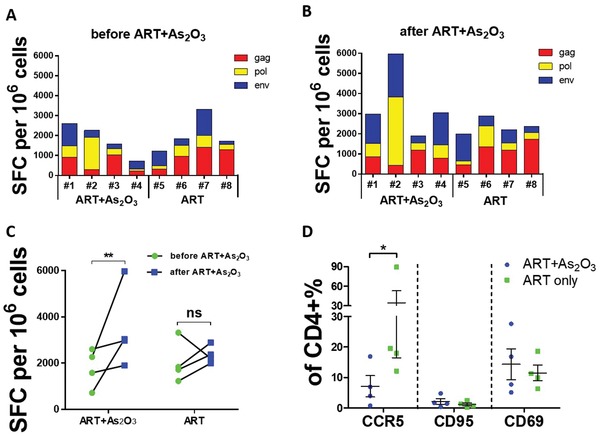
Enhanced SIV specific T cell immune responses after administration of arsenic trioxide in combination with ART in chronic SIV‐infected rhesus macaques. Specific T cell immune responses against the SIVmac239 Gag, Pol, and Env antigens were monitored by IFN‐γ‐mediated ELISPOTs at different time points: A) before ART in combination with arsenic trioxide therapy; B) after ART in combination with arsenic trioxide therapy. C) Statistical analysis of the ELISPOT data before and after arsenic trioxide treatment. D) Effect on T cell activation markers, including CCR5, CD95, and CD69 after administration of arsenic trioxide four weeks in SIV‐infected macaques. SFC, spot forming cells. The final data were represented as the means ± standard deviations of triplicate experiments. *p < 0.05, **p < 0.01.

In the present study, we found that arsenic class of mineral, arsenic trioxide, a drug for the clinical treatment of APL and HCC, can reactivate the latent viral reservoir in vitro and in vivo. The therapeutic effect of arsenic trioxide was explored for the first time in a model of chronically SIV‐infected macaques. In combination with ART, arsenic trioxide can significantly reduce the viral reservoirs and delay viral rebound after ART termination in this macaque model. Based on our current results, the likely mechanism for delaying viral rebound by this regiment may involve with at least three actions, including perturbation of viral reservoir, downregulation of CD4 primary receptors and CCR5 coreceptors for SIV entry on CD4+ T cells, and enhancement of SIV specific immune responses.

As a prerequisite for HIV functional cure, we first verified how arsenic trioxide impacted the latent proviruses. Our results demonstrated that it effectively reactivated viral transcription and replication not only in J‐Lat HIV latency cell line but in primary CD4+ T cells from chronically SIV‐infected macaques and HIV‐infected patients. Arsenic trioxide is able to regulate multiple transcriptional signaling factors, including NF‐kB, protein kinase C, PML‐NB, etc., and these signaling pathways are actually associated with the reactivation by other LRAs such as bryostatin‐1 and SAHA.^30^, ^31^, ^32^ Notably, this reactivation by arsenic trioxide treatment was not accompanied by significantly devastating T cell activation and proinflammatory responses (Figures 2 and 3). Despite the underlying mechanisms remain to be determined, the characteristic of arsenic trioxide is superior to other LRAs. Nevertheless, the ultimate answer should come from future clinical studies in HIV‐infected patients.

Intriguingly, we found that arsenic trioxide downregulated cell surface expression of CD4 and CCR5, which are the key receptors and coreceptors to mediate HIV‐1/SIV attachment and entry into target cells. It is worth noting that the two successful cases of HIV cure (Berlin patient and London patient) are attributed to the use of CCR5‐△32 mutant bone marrow transplants,^33^, ^34^ which illustrated the importance of CCR5 receptor in controlling HIV infection. Our current research, downregulating CCR5 expression in vitro (Figure 5) and in SIV‐infected macaques (Figure 6) by arsenic trioxide treatment, shares somehow analogical characteristic with these two encouraging examples. Thus, arsenic trioxide may prevent the spread of viral infection to bystander CD4+ T cells. However, it is unclear to what degree the downregulation of CD4 and CCR5 contributes to the regulation of viral infection in the presence of arsenic trioxide and ART. Interestingly, recent studies demonstrated that some potential LRAs, such as Bryostatin‐1, PEP005, and diterpene analogs were capable of reducing the HIV receptors and coreceptors CD4, CCR5, and CXCR4 expression and the susceptibility of naive CD4+ T cells to HIV‐1 acquisition.^35^, ^36^, ^37^ The mechanisms of downregulating the HIV receptor/coreceptor by some LRAs remain to be investigated. However, these results suggest that a novel strategy for functionally curing HIV infection might be developed by reducing or inhibiting the expression of CCR5 receptor on the surface of CD4+ cells by drug treatment.

Based on our observation, we proposed that the antigen‐specific immune responses should ideally be improved simultaneously when an LRA is used in order to effectively eliminate those activated viruses. The importance of antigen‐specific immune responses in controlling HIV/SIV infection has been extensively emphasized by many studies.^38^, ^39^, ^40^, ^41^ For example, off‐therapy remission of SIV viremia after ART discontinuation was observed when SIV‐infected Indian rhesus macaques were treated using buthionine sulfoximine in combination with auranofin, and this control was correlated with the enhanced antigen‐specific CD8+ T cells.^42^ To some extent, the mechanism of arsenic trioxide controlling viral rebound in this study is similar to that of above drugs. That is, SIV‐specific immune responses were significantly increased by arsenic trioxide treatment, which play important role in eliminating the infected cells harboring latent SIV during the viral reactivation. The enhancement of SIV‐specific immune responses by arsenic trioxide treatment, therefore, contributed to suppressing viral rebound in chronic SIV‐infected rhesus macaques.

Our study has limitations. This is a pilot study of relatively small numbers of macaques, and only four animals per group were used in this study. Future studies with a larger number of macaques, in both acute and chronic infection models, should be performed with arsenic trioxide in combination with ART. Although the number of macaques was relatively limited, these eight macaques were distributed very carefully based on age, sex, weight, and genotype. All SIV‐infected macaques used in this study had a long history of SIV infection and had several courses of ART therapy, which mimics treatment of chronic HIV‐1 infection in humans. These macaques were infected with SIVmac239 for more than 5 years, and the viral load in plasma ranged from 3.60 to 6.33 log when starting this study (Table S1, Supporting Information). Highly pathogenic SIV‐infected macaques have been well‐validated as a stringent model to recapitulate HIV‐1 pathogenesis and persistence during ART therapy in humans.^43^ Indeed, in our Chinese rhesus model, ART treatment effectively suppressed SIV infection to undetectable levels in plasma, and upon ART discontinuation, virus rapidly rebounded, which is very similar with that in ART‐treated HIV patients. Previous studies showed that SIV pathogenesis in Chinese‐origin macaques resembled HIV‐1 infections in adult humans more closely, compared with that in Indian‐origin macaques.^43^, ^44^, ^45^ Although arsenic trioxide in combination with ART demonstrated a promising result in our animal model, future studies are needed to test if repeated courses of arsenic trioxide with ART might progressively deplete latent viral reservoir.

Taken together, our study demonstrate that arsenic trioxide effectively reactivates viral latency and delays viral rebound after termination of ART in chronically SIV‐infected rhesus macaques. The mechanism may involve with multiple modes acting synergistically. Arsenic trioxide inhibits the spread of released SIV due to reactivation of latent SIV provirus by downregulating the expression of CD4 receptors and CCR5 coreceptors of host cells, which reduce susceptibility of virus to attach and enter CD4+ T cells. Furthermore, suppression of SIV infection and replication can be enhanced by the restored SIV specific immune responses. Since arsenic trioxide formulation is commercially available and being approved as a drug for the treatment of malignancies in humans, further studies should consider combining arsenic trioxide with ART in HIV‐1‐infected patients.

## Experimental Section

*Drugs and SIV Peptide Pools*: Arsenic trioxide (Beijing ShuangLu Pharmaceutical Co., Ltd., China) was a clinically available drug for treatment of APL and advanced HCC, which was issued by China Food and Drug Administration (approval number: H20080665).

*Reverse Transcriptase Inhibitors*: PMPA (also called tenofovir) and FTC (also called emtricitabine) were provided by Shanghai Desano Pharmaceutical Co., Ltd. Those drugs were provided in powder formation of active pharmaceutical ingredient, and dissolved in 0.9% saline solution to a final concentration as below.

Peptide pools, which covered the entire SIVmac239 sequences of Gag, Pol, Env, Nef, Vif, Vpx, Vpr, Rev, and Tat proteins, were obtained through the AIDS Research and Reference Reagent Program, National Institutes of Health (NIH), USA. Peptide pools consisted of 15 amino acids shifted by 11 overlapping amino acids residues, and dissolved in dimethyl sulfoxide to a final concentration of 0.4 mg per peptide mL^−1^ before use.

*CD4+ T Cell Sorting, Reactivation, RNA Extraction, and Nested Real‐Time Polymerase Chain Reaction (PCR) Assay for Cell‐Associated RNA Detection*: PBMCs from HIV‐infected subjects or SIV‐infected macaques were isolated by standard Ficoll‐Hypaque density gradient centrifugation. CD4+ T cells were sorted using a human CD4+ T cell isolation kit or nonhuman primate CD4+ T cell isolation kit (Miltenyi Biotec), following the standard magnetic cell sorting protocol. The purified CD4+ T cells were counted and suspended in 200 µL of medium (Roswell Park Memorial Institute (RPMI) 1640 with 10% fetal bovine serum (FBS) and 1% penicillin/streptomycin) in 96‐well U‐bottom plate. Each well had 0.5 to 2 million of CD4+ T cells. After 24 h stimulation with different compounds, cell‐associated RNA was extracted according to the manufacturer's protocol (Qiagen, RNeasy Mini Kit), and eluted into 30 µL of RNase‐free water. Aliquots of 15 µL RNA were directly reverse‐transcribed following the standard procedure (BIO‐RAD, transcription supermix for reverse transcription‐quantitative real‐time PCR (RT‐qPCR)). For nested real‐time PCR, cell‐associated complementary DNA (cDNA) or serial dilutions of cDNA standards was subjected to two rounds of PCR amplification.

Two kinds of cell‐associated RNA were detected, including msRNA (multiply spliced transcripts of HIV/SIV) and usRNA (unspliced transcripts of HIV/SIV). For detection of msRNA, three primers crossing Tat and Rev for seminested real‐time PCR were designed, because of the sequence specificity of the detection region. The first round of msRNA PCR was performed with the primer msRNA‐1F and msRNA‐R, and the second‐round real‐time PCR was performed with the primer pair msRNA‐2F and msRNA‐R. Either Taqman probes or SYBR Green were used for msRNA detection at the second‐round real‐time PCR, and the amplicon sizes were 130 bp for the first‐round PCR and 103 bp for the second‐round real‐time PCR. For detection of usRNA, two pairs of primers were designed to amplify a region within the HIV‐1/SIV gag gene. The primer pair U1‐5F and U1‐3R was used in the first‐round PCR and the amplicon size was 227 bp. The primer pair U2‐5F and U2‐3R was used in the second‐round PCR, and the amplicon size was 176 bp. All primer sequences used in this study were listed in Table S3, Supporting Information.

*Quantitative PCR Analysis of Cytokines*: PBMCs from healthy or SIV‐infected macaques were isolated using Ficoll‐Hypaque solution, and incubated with arsenic trioxide overnight. The total RNA was extracted from cultured cells using a RNA easy Mini Kit (Qiagen) and reverse‐transcribed with iScript cDNA synthesis kit (Bio‐Radioquantitative PCR was performed with a QuantiFast SYBR Green PCR Kit (Qiagen) in the CFX‐96 Real‐time PCR system (Bio‐Rad). β‐macroglobulin RNA was used as the internal control. A cocktail of PMA and ionomycin was used as a positive control for T cell activation. All primer sequences used in this study were listed in Table S3, Supporting Information.

*Animals and Treatment*: Chinese rhesus monkeys (Macaca mulatta) were housed in the Experimental Animal Center of Guangzhou Institutes of Biomedicine and Health (GIBH, Guangzhou, China). The use of animals in this study was approved by our Institutional Animal Care and Use Committee. All monkeys in this study were chronically infected with SIVmac239 and assigned into two groups based on comparable viral load, weight, and sex (Table S1, Supporting Information). They received the following antiretroviral therapy for 12 weeks: 30 mg kg^−1^ PMPA and 20 mg kg^−1^ FTC, both injected subcutaneously once daily. During ART treatment, four monkeys received 0.16 mg kg^−1^ arsenic trioxide in 0.9% saline through intravenous infusion once daily for four weeks. Another group of four monkeys were used as controls with mock treatment. They received the same volume of saline through intravenous infusion once daily for four weeks.

*Immunological Assays*: Immunological assays included the ELISPOT assay, intracellular cytokine staining (ICS) for polyfunctionality of T lymphocytes and CFSE staining for cell proliferation; these were performed as described previously.^27^, ^38^ Multicolor ICS assays and phenotypic markers of T lymphocyte population were performed with the following monoclonal antibodies: anti‐CD3‐Pacific Blue, anti‐CD4‐AmCyan, anti‐CD8‐allophycocyanin (APC)‐Cy7, anti‐CD28‐fluorescein isothiocyanate (FITC), anti‐CD95‐phycoerythrin (PE)‐Cy5, anti‐IFN‐PE, anti‐TNF‐α‐PE‐Cy7, and anti‐IL‐2‐APC (BD Pharmingen). Samples were analyzed with a FACSAria flow cytometer (BD Biosciences) and FlowJo software (version 7.6; Tree Star, Inc.). The number of circulating CD4+ T lymphocytes was determined using BD TruCount tubes according to the manufacturer's instructions (BD Biosciences). Activation markers were detected with the following monoclonal antibodies: anti‐CD3‐PerCP, anti‐CD4‐FITC, anti‐CD4‐APC, anti‐CD38‐FITC, anti‐CD25‐APC, anti‐CD69‐PE, anti‐CD95‐PE‐Cy5, anti‐Ki67‐PE, anti‐CCR5‐PE. Samples were analyzed with a Accuri C6 flow cytometer (BD Biosciences) and FlowJo software (version 7.6; Tree Star, Inc.).

*Viral Load Determination by Real‐Time PCR*: Levels of plasma SIV RNA were quantitated by real‐time PCR as described previously.^27^, ^38^ Briefly, viral RNA was isolated from plasma using the QIAamp Viral RNA Minikit (Qiagen) and amplified using the QuantiTect SYBR Green RT‐PCR Kit (Qiagen). Primers were designed to match the SIVmac239 gag sequence. The copy number of viral RNA was calculated based on the standard curve of an in vitro‐transcribed fragment of the SIVmac239 gag gene. The limitation for this assay was 100 copies mL^−1^ plasma.

*Viral DNA Assays*: Levels of integrated SIVmac239 DNA in PBMCs were quantitated using Alu‐PCR method, as previously described.^38^, ^46^ Briefly, PBMCs from the macaques were isolated by standard Ficoll‐Hypaque density gradient centrifugation, and CD4+ T cells were purified using a nonhuman primate CD4+ T cell isolation kit (MiltenyiBiotec), following the standard magnetic cell sorting protocol. The purified CD4+ T cells were counted, and total cellular DNA was isolated from 5 × 10^6^ cells using a QIAamp DNA Blood Mini kit (Qiagen). The integrated SIVmac239 DNA was determined by nested PCR with two pairs of primers. A mix with SIVgag‐reverse primer and Alu‐forward primer were used in the first round of PCR, and a pair of primers specific to a conserved region of SIVgag gene was used in the second round of PCR. Quantification was analyzed by comparing with an integration standard of CEMss/pWPXLD‐rc cell genome. PCR assays were performed with 100–500 ng samples of DNA.

*Quantitation of Circulating CD4+ T Lymphocytes*: The number of circulating CD3+, CD4+, and CD8+ T lymphocytes were determined using BD TruCount tubes according to the manufacturer's instructions (BD Biosciences).

*Statistics*: Flow cytometry software analysis was performed using FlowJo 7.6 (Tree Star Inc.). Graphical representations were generated with GraphPad Prism 5.01 (GraphPad Software Inc., La Jolla, CA). Two‐tailed *p* values were calculated for all analyses, and differences were considered statistically significant when *p* values were less than 0.05.

## Conflict of Interest

The authors declare no conflict of interest.

## Supporting information

Supplementary
