# Supplementary material for: Arsenic Trioxide Impacts Viral Latency and Delays Viral Rebound after Termination of ART in Chronically SIV‐Infected Macaques
Source: Adv Sci (Weinh). 2019 May 7;6(13):1900319. doi: 10.1002/advs.201900319 (PMC6662089; doi:10.1002/advs.201900319)
Supplement: Supplementary file 1 — Supplementary [file ADVS-6-1900319-s001.pdf]

Copyright WILEY-VCH Verlag GmbH & Co. KGaA, 69469 Weinheim, Germany, 2019.

ADVANCED SCIENCE
Open Access

Supporting Information

for *Adv. Sci.,* DOI: 10.1002/advs.201900319

Arsenic Trioxide Impacts Viral Latency and Delays Viral Rebound after Termination of ART in Chronically SIV-Infected Macaques

Qing Yang, Fengling Feng, Pingchao Li, Enxiang Pan, Chunxiu Wu, Yizi He, Fan Zhang, Jin Zhao, Ruiting Li, Liqiang Feng, Fengyu Hu, Linghua Li, Huachun Zou, Weiping Cai, Thomas Lehner, Caijun Sun,* and Ling Chen**

Copyright WILEY-VCH Verlag GmbH & Co. KGaA, 69469 Weinheim, Germany, 2019.

Supporting Information

Arsenic trioxide regulated viral reservoir in chronically SIV infected macaques

Qing Yang, Fengling Feng, Enxiang Pan, Pingchao Li, Chunxiu Wu, Yizi He, Fan Zhang, Jin Zhao, Ruiting Li, Liqiang Feng, Fengyu Hu, Linghua Li, Huachun Zou, Weiping Cai, and Thomas Lehner, Caijun Sun*, Ling Chen**

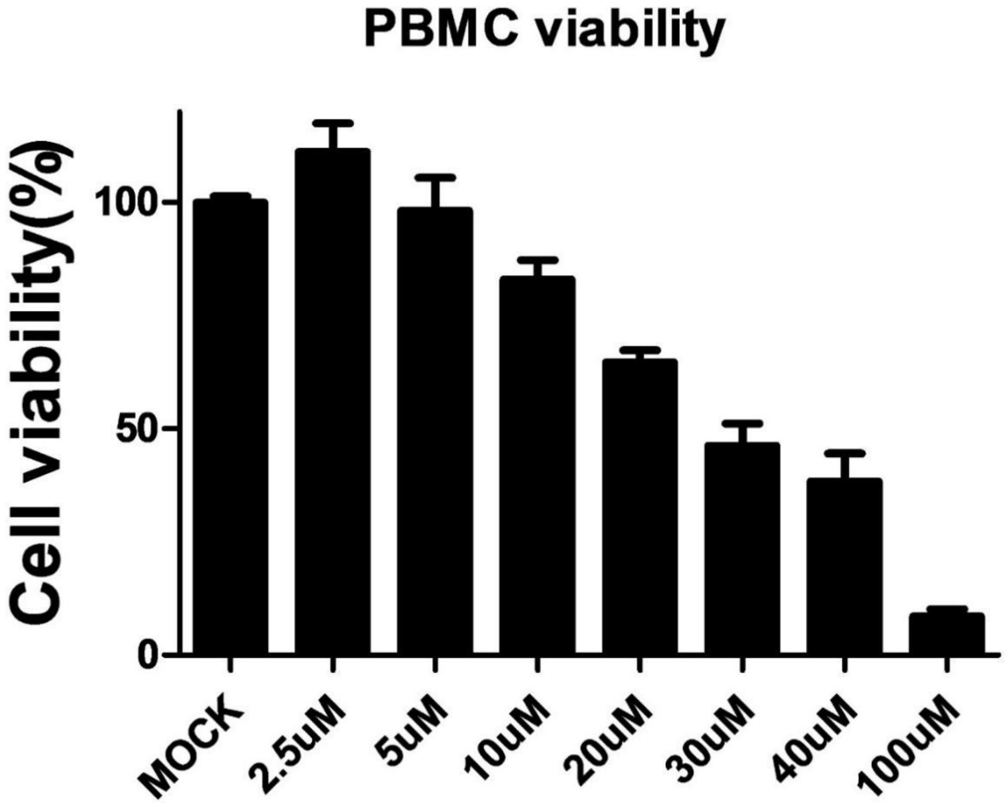

Figure S1. Cytotoxicity on rhesus macaque PBMCs by arsenic trioxide treatment. Macaques PBMCs were treated with arsenic trioxide with the given concentrations for 24hrs and the cell viability was then measured by CCK-8 kit (Dojindo). Data show the means ± standard deviations in three independent experiments.

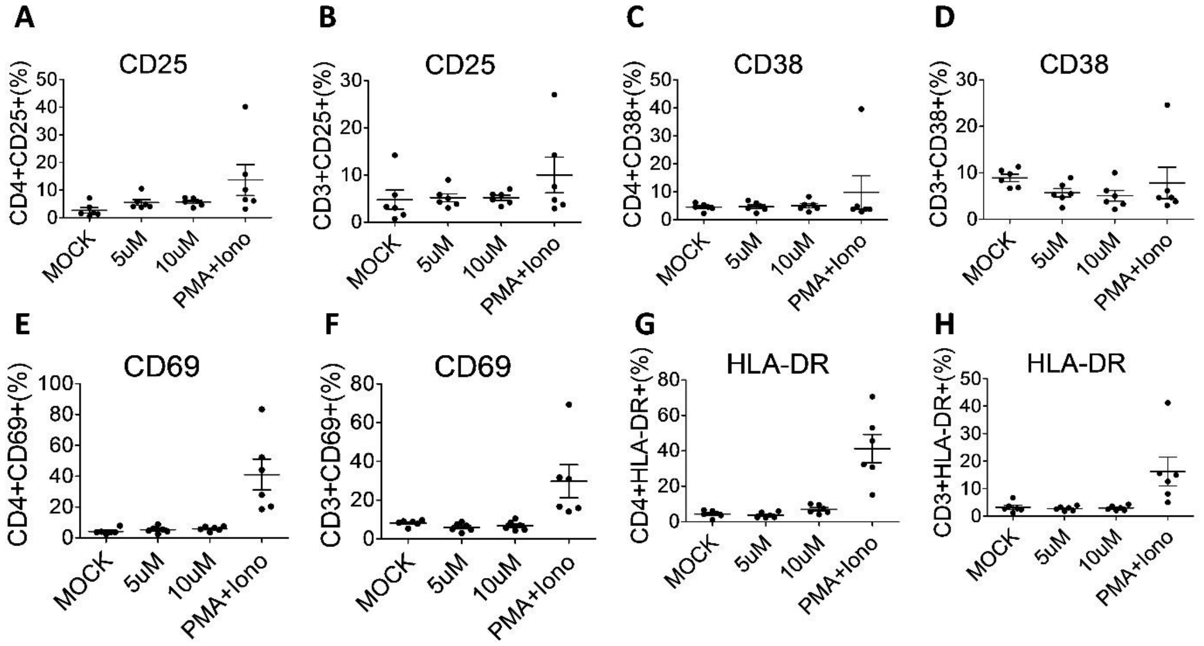

Figure S2. T cell activation and proliferation after arsenic trioxide treatment (5 days) in SIV-infected macaque's PBMC. PBMCs from SIV-infected macaques were treated with arsenic trioxide for 5 days. The expressions of T cell activation markers were detected by flow cytometry, including CD25+ CD69+ and CD38+/HLA-DR+ on the surface of CD3+ T A), C), E), G) and CD4+ T cells B), D), F), H). A cocktail of PMA and ionomycin was used as a positive control for T cell activation.

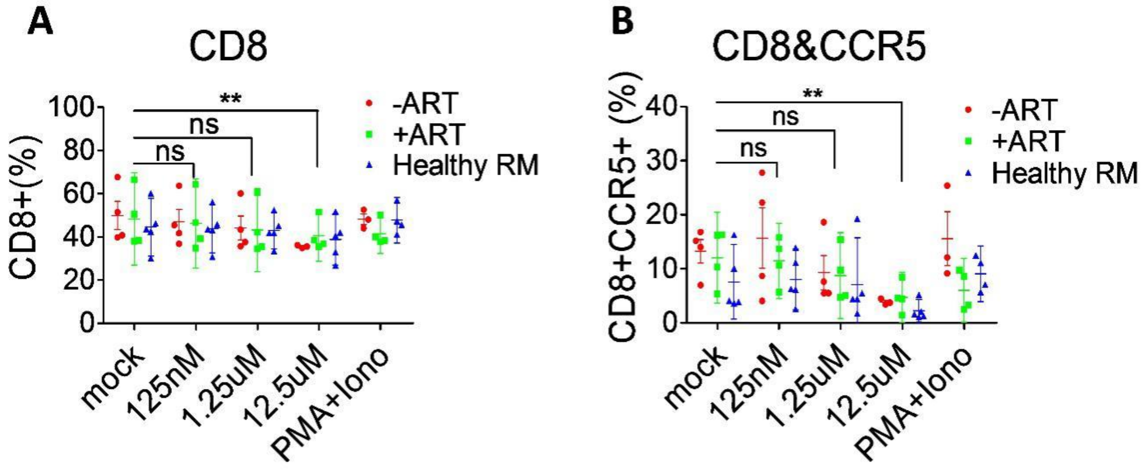

Figure S3. Changes of CD8 and CCR5 on the surface of CD8+T cells after arsenic trioxide treatment. Primary PBMCs isolated from SIV-infected or SIV-negative healthy macaques

were treated with arsenic trioxide for 5 days, and the proportion of CD8+ T cells A), the expression level of CCR5 on CD8+T cell surface B) were assessed using flow cytometry.

Table S1. Characterization of rhesus macaques used in this study.

Group	Animal Number	Animal ID	Sex	Weight (Kg)	Age (year)	Infection History (year)	Viral load before study (log10/ml)
ART+ As_2O_3	#1	04149	M	8.66	10	5	3.6
	#2	021859	M	7.49	12	6	6.33
	#3	031496	F	5.66	11	6	3.85
	#4	03058	F	5.03	11	5	5.43
ART+ saline	#5	031137	M	9.18	11	5	3.75
	#6	031517	M	9.16	11	6	5.89
	#7	04086	F	5.29	10	5	3.76
	#8	04198	F	4.99	10	5	5.51

Table S2. Characterization of HIV patients for this study

Patient Number	Sex	Age (year)	ART start time	ART regimen	CD4 count before ART (/ul)	CD4 count after ART (/ul)	Viral load (copies/ ml)
#1	M	55	2015/4/17	TDF+3TC+EFV	385	455	<20
#2	M	39	2015/5/19	TDF+3TC+EFV	272	605	<20
#3	M	51	2011/3/1	AZT+3TC+NVP	194	357	<20
#4	M	35	2015/7/1	TDF+3TC+EFV	314	454	<20

Table S3. Sequence of primer and probe used in this study

Symbol of Primer or probe		Type`	Sequence (5'-3')
SIV msRNA	SIV MS-1F	Sense	GAAGAAGAACTCCGAAAAAGG
	SIV MS-R	Anti-sense	GCTGTTGCCACCGCC
	SIV MS-2F	Sense	CTAATACATCTTCTGCATCAAAC
	SIV MS-FAM	probe	FAM-ATATCCAACAGGACCCGGC-TAMRA
SIV usRNA	SIV US-1F	Sense	AAATACTTTCGGTCTTAGCTTCCATT
	SIV US-1R	Anti-sense	TAATTTCCTCCTCTGCCGCTAG
	SIV US-2F	Sense	CATTAGTGCCAACAGGCTCAGA
	SIV US-2r	Anti-sense	CTTGGTCCACTTGTTTTTGGCATAG
	SIV US-Cy5	probe	Cy5-CTGTTGTTCCTGTTTCCACCACTAGGTGTC-BHQ2
HIV msRNA	HIV MS-1F	Sense	CTTAGGCATCTCCTATGGCAGGAA
	HIV MS-R	Anti-sense	GGATCTGTCTCTGTCTCTCTCTCCACC
	HIV MS-2F	Sense	ACAGTCAGACTCATCAAGTTTCTCTATCAAAGCA
	HIV MS-FAM	probe	FAM-TTCCTTCGGGCCTGTCGGGTCCC-BHQ1
HIV usRNA	HIV US-F	Sense	TCAGCCCAGAAGTAATACCCGAGT
	HIV US-1R	Anti-sense	TGCTATGTCAGTTCCCCTTGGTTCTCT
	HIV US-2R	Anti-sense	CACTGTGTTTAGCATGGTGTTT
	HIV US-Cy5	probe	Cy5-ATTATCAGAAGGAGCCACCCCACAAGA-BHQ2
IL-1 β		Sense	ACGTCGATGGCCCTAAACAG
		Anti-sense	AAGCCCTCGTTGTAGTGCTC
IL-6		Sense	CCAGTACTCCCAGGAGAAGATTC
		Anti-sense	GTCGAGGATGTACCGAATGTGT
IL-8		Sense	CTCCAAACCTTTCCACCCCA
		Anti-sense	CTCTGCACCCATGGTTCCTT
IFN-γ		Sense	AGAGTGTGGAGACCATCAAGGA
		Anti-sense	TGCGTTGGACATTCGAGTCAG
TNF-α		Sense	GAGCACTGAAAGCATGATCCG
		Anti-sense	GGAGAAGAGGCTGAGGAACCA
